# Sperm and leukocyte telomere length are related to sperm quality parameters in healthy men from the Led-Fertyl study

**DOI:** 10.1093/hropen/hoae062

**Published:** 2024-10-14

**Authors:** María Fernández de la Puente, Cristina Valle-Hita, Albert Salas-Huetos, María Ángeles Martínez, Elena Sánchez-Resino, Silvia Canudas, Daniel Torres-Oteros, Joana Relat, Nancy Babio, Jordi Salas-Salvadó

**Affiliations:** Universitat Rovira i Virgili, Departament de Bioquímica i Biotecnologia, Alimentació, Nutrició, Desenvolupament i Salut Mental (ANUT-DSM), Unitat de Nutrició Humana, Reus, Spain; Consorcio CIBER, M.P. Fisiopatología de la Obesidad y Nutrición (CIBERObn), Instituto de Salud Carlos III (ISCIII), Madrid, Spain; Institut d’Investigació Sanitària Pere Virgili (IISPV), Reus, Spain; Universitat Rovira i Virgili, Departament de Bioquímica i Biotecnologia, Alimentació, Nutrició, Desenvolupament i Salut Mental (ANUT-DSM), Unitat de Nutrició Humana, Reus, Spain; Consorcio CIBER, M.P. Fisiopatología de la Obesidad y Nutrición (CIBERObn), Instituto de Salud Carlos III (ISCIII), Madrid, Spain; Institut d’Investigació Sanitària Pere Virgili (IISPV), Reus, Spain; Consorcio CIBER, M.P. Fisiopatología de la Obesidad y Nutrición (CIBERObn), Instituto de Salud Carlos III (ISCIII), Madrid, Spain; Institut d’Investigació Sanitària Pere Virgili (IISPV), Reus, Spain; Universitat Rovira i Virgili, Departament de Bioquímica i Biotecnologia, Alimentació, Nutrició, Desenvolupament i Salut Mental (ANUT-DSM), Unitat de Medicina Preventiva, Reus, Spain; Department of Nutrition, Harvard T.H. Chan School of Public Health, Boston, MA, USA; Universitat Rovira i Virgili, Departament de Bioquímica i Biotecnologia, Alimentació, Nutrició, Desenvolupament i Salut Mental (ANUT-DSM), Unitat de Nutrició Humana, Reus, Spain; Consorcio CIBER, M.P. Fisiopatología de la Obesidad y Nutrición (CIBERObn), Instituto de Salud Carlos III (ISCIII), Madrid, Spain; Institut d’Investigació Sanitària Pere Virgili (IISPV), Reus, Spain; Department of Pharmacology, Therapeutics and Toxicology, Faculty of Veterinary, Universitat Autonòma de Barcelona, Cerdanyola del Vallès, Catalonia, Spain; Laboratory of Toxicology and Environmental Health, School of Medicine, Universitat Rovira i Virgili, IISPV, Reus, Catalonia, Spain; Center of Environmental, Food and Toxicological Technology—TecnATox, Universitat Rovira i Virgili, Reus, Spain; Department of Nutrition, Food Sciences and Gastronomy, School of Pharmacy and Food Sciences, Food Torribera Campus, University of Barcelona, Santa Coloma de Gramenet, Spain; Institute of Nutrition and Food Safety of the University of Barcelona, INSA-UB Maria de Maeztu Unit of Excellence, Santa Coloma de Gramenet, Spain; Department of Nutrition, Food Sciences and Gastronomy, School of Pharmacy and Food Sciences, Food Torribera Campus, University of Barcelona, Santa Coloma de Gramenet, Spain; Institute of Nutrition and Food Safety of the University of Barcelona, INSA-UB Maria de Maeztu Unit of Excellence, Santa Coloma de Gramenet, Spain; Consorcio CIBER, M.P. Fisiopatología de la Obesidad y Nutrición (CIBERObn), Instituto de Salud Carlos III (ISCIII), Madrid, Spain; Department of Nutrition, Food Sciences and Gastronomy, School of Pharmacy and Food Sciences, Food Torribera Campus, University of Barcelona, Santa Coloma de Gramenet, Spain; Institute of Nutrition and Food Safety of the University of Barcelona, INSA-UB Maria de Maeztu Unit of Excellence, Santa Coloma de Gramenet, Spain; Universitat Rovira i Virgili, Departament de Bioquímica i Biotecnologia, Alimentació, Nutrició, Desenvolupament i Salut Mental (ANUT-DSM), Unitat de Nutrició Humana, Reus, Spain; Consorcio CIBER, M.P. Fisiopatología de la Obesidad y Nutrición (CIBERObn), Instituto de Salud Carlos III (ISCIII), Madrid, Spain; Institut d’Investigació Sanitària Pere Virgili (IISPV), Reus, Spain; Universitat Rovira i Virgili, Departament de Bioquímica i Biotecnologia, Alimentació, Nutrició, Desenvolupament i Salut Mental (ANUT-DSM), Unitat de Nutrició Humana, Reus, Spain; Consorcio CIBER, M.P. Fisiopatología de la Obesidad y Nutrición (CIBERObn), Instituto de Salud Carlos III (ISCIII), Madrid, Spain; Institut d’Investigació Sanitària Pere Virgili (IISPV), Reus, Spain

**Keywords:** telomere length, sperm quality, male infertility, spermatozoa, leukocyte, reproductive age, Led-Fertyl study

## Abstract

**STUDY QUESTION:**

Could sperm and leukocyte telomere length (TL) be associated with sperm quality parameters and reproductive health in men from the general population?

**SUMMARY ANSWER:**

A positive association between sperm and leukocyte TL with sperm concentration and total count has been demonstrated.

**WHAT IS KNOWN ALREADY:**

Male factors account for almost half of cases of couple infertility, and shorter TLs have been observed in sperm from men with impaired sperm parameters. However, evidence in men from the general population is limited.

**STUDY DESIGN, SIZE, DURATION:**

A total of 200 volunteers of reproductive age were recruited between February 2021 and April 2023 to participate in the Lifestyle and Environmental Determinants of Seminogram and Other Male Fertility-Related Parameters (Led-Fertyl) cross-sectional study.

**PARTICIPANTS/MATERIALS, SETTING, METHODS:**

TLs in sperm and leukocytes were measured using quantitative polymerase chain reaction (qPCR) in 168 and 194 participants, respectively. Sperm parameters, including concentration, total count, motility, vitality, and morphology, were analyzed using a computer-assisted sperm analysis (CASA) SCA^®^ system according to the World Health Organization (WHO) 2010 guidelines. Multivariable regression models were performed to assess the associations between sperm and leukocyte TL, either in tertiles or as continuous variables, and sperm quality parameters while adjusting for potential confounders.

**MAIN RESULTS AND THE ROLE OF CHANCE:**

Participants in tertiles 2 (T2) and 3 (T3) of sperm TL showed a higher sperm concentration (β: 1.09; 95% CI: 0.09–2.09 and β: 2.06; 95% CI: 1.04–3.09 for T2 and T3, respectively; *P*-trend < 0.001), compared to those in the reference tertile (T1). Participants in the highest tertile of sperm TL showed higher total sperm count (β: 3.83; 95% CI: 2.08–5.58 for T3 vs T1; *P*-trend < 0.001). Participants in the top tertile of leukocyte TL showed higher sperm concentration (β: 1.49; 95% CI: 0.44–2.54 for T3 vs T1; *P*-trend = 0.004), and total count (β: 3.49; 95% CI: 1.62–5.35 for T3 vs T1; *P*-trend < 0.001) compared with participants in T1. These results remained consistent when sperm and leukocyte TL were modelled as continuous variables.

**LIMITATIONS, REASONS FOR CAUTION:**

One limitation is the impossibility of establishing a cause–effect relationship due to the cross-sectional study design. Additionally, the sample size of the study cannot be considered large.

**WIDER IMPLICATIONS OF THE FINDINGS:**

Sperm and leukocyte TLs are associated with sperm quality parameters in the general population. Additional determinations and further studies with larger sample sizes are needed to clarify the mechanisms underlying these associations and to investigate the further implications.

**STUDY FUNDING/COMPETING INTEREST(S):**

The Led-Fertyl study was supported by the Spanish government’s official funding agency for biomedical research, Instituto de Salud Carlos III (ISCIII), through the Fondo de Investigación para la Salud (FIS) and co-funded by the European Union ERDF/ESF, ‘A way to make Europe’/‘Investing in your future’ (PI21/01447), and the Diputació de Tarragona (2021/11-No.Exp. 8004330008-2021-0022642). J.S.-S., senior author of the present study, is partially supported by ICREA under the ICREA Academia program. M.F.d.l.P. was supported by a predoctoral grant from the Rovira i Virgili University and Diputació de Tarragona (2020-PMF-PIPF-8). C.V.-H. received a predoctoral grant from the Generalitat de Catalunya (2022 FI_B100108). M.Á.M. was supported by the Sara Borrell postdoctoral fellowship (CD21/00045—Instituto de Salud Carlos III (ISCIII)). All authors declare that they have no conflicts of interest.

**TRIAL REGISTRATION NUMBER:**

N/A.

WHAT DOES THIS MEAN FOR PATIENTS? The proportion of the global population who have experienced infertility was reported as 17.5% in 2020 and male factors are responsible for almost half of cases of couple infertility. In recent years, the study of the mechanisms related to reproductive health has become essential due to the impact of infertility on mental health and quality of life.

In this sense, telomeres, which are specialized protective regions at the end of chromosomes, have been previously proposed as biomarkers of sperm quality and male infertility since short telomere lengths have been found in men with impaired sperm parameters. However, most of these published articles have not included men from the general population and have instead used data from men attending fertility clinics.

To increase evidence in this field, we conducted a cross-sectional study including 200 healthy volunteer participants of reproductive age. We found that telomere lengths in sperm and in white blood cells are positively associated with sperm concentration and total count, independent of age, body mass index, and other potential confounding factors. Additional studies are needed to reproduce and elucidate the mechanisms underlying these associations.

## Introduction

Total fertility rate, reported as the number of births per woman, has drastically decreased worldwide between the 1950s and 2022 (United Nations, 2022) and the estimated proportion of the population who have experienced infertility during their lifetime reached 17.5% in 2020 (World Health Organization, 2023), with male factors accounting for almost half of the cases of couple infertility (Agarwal *et al.*, 2021). The causes of male infertility are multifactorial, with varicocele being one of the most common, followed by endocrine, metabolic, and genetic disorders. These conditions, in association with environmental factors, may reduce semen quality (Tahmasbpour *et al.*, 2014). Given the significant impact on the mental health and quality of life of the population, investigations of the underlying mechanisms related to reproductive health are essential (Lei *et al.*, 2021).

The nucleoprotein complexes that protect and maintain genome integrity are known as telomeres (Blackburn, 1991). These structures, located at the end of eukaryotic chromosomes, consist of the repetition in tandem of the DNA sequence 5′-TTAGGG-3′ and a specialized multiprotein complex, called Shelterin (de Lange, 2005). In somatic cells and under typical conditions, a small fragment of telomeric DNA is shortened after each cell division due to problems with end-replication (Shammas, 2011). In male germ cells, telomeres preserve and increase their length over time to ensure genome integrity across generations. Mature spermatozoa, in fact, show longer telomere length (TL) when compared to somatic cells (Rocca *et al.*, 2019).

Over the past few years, the relationship between TL and sperm quality has been investigated. Data from a recent systematic review and meta-analysis (SRMA) of case-control studies (Fernández de la Puente *et al.*, 2024) showed that individuals diagnosed with infertility had shorter sperm and leukocyte TL compared to fertile controls. Furthermore, it was observed that TL in spermatozoa is also shorter in men with low sperm concentration or total count, compared to men with normal sperm quality parameters (Fernández de la Puente *et al.*, 2024). However, in that SRMA, the cross-sectional studies reviewed reported contradictory results. The majority of the articles included in this SRMA were conducted in men from couples attending fertility clinics or those included in assisted reproduction programs, but few studies were conducted in men from the general population. Additionally, these studies did not consider demographic characteristics or lifestyle factors that have been shown to modulate the relationship between TL and sperm quality, such as age, energy intake, smoking habits, physical activity (Gürel *et al.*, 2024), body mass index (BMI) (Raee *et al.*, 2023; Moustakli *et al.*, 2024), or nutrition (Galiè *et al.*, 2020).

Therefore, the main aim of this study was to assess the associations between sperm and leukocyte TL and sperm quality outcomes in healthy volunteers of reproductive age from the Lifestyle and Environmental Determinants of Seminogram and Other Male Fertility-Related Parameters (Led-Fertyl) study. The main hypothesis is that longer sperm and leukocyte TL are positively associated with better parameters of sperm quality.

## Materials and methods

### Study design

The Led-Fertyl study is a cross-sectional study aimed to identify the associations between lifestyle and environmental factors and sperm quality and to explore potential mechanisms elucidating these associations. The principal objective of the present analysis was to explore the potential associations between sperm and leukocyte TL and sperm quality parameters.

### Study participants

A total of 200 volunteers were enrolled in the Led-Fertyl study from February 2021 to April 2023. The recruitment process was conducted through the dissemination of information at the Rovira i Virgili University and in various towns and villages across Catalonia (Spain). In addition, flyers and posters were exhibited in establishments, pharmacies, hospitals and primary healthcare centers, and through social media and newspapers. The main inclusion criteria were men aged between 18 and 40 years with good health status and registered in the Spanish public health system. Exclusion criteria were: inability to follow scheduled intervention visits; institutionalization; history of intestinal resection or the presence of inflammatory bowel disease; history of major organ transplantation; known reproductive disease or vasectomy; documented history of cardiovascular disease; concurrent therapy with immunosuppressive drugs, cytotoxic agents, systemic corticosteroids, or medications associated with sperm disorders; excessive weight loss (over 5 kg in the last month); presence of cirrhosis or liver failure; endocrine diseases; immunodeficiency disorders, hepatitis B or C; alcoholism or drug abuse; active malignant cancer or a history of malignancy within the past 5 year; severe psychiatric disorder; and any condition of severe comorbidity with <24 months of life expectancy, or any other condition that may interfere with compliance with the study protocol. Once enrolled in the study, participants were asked to complete online self-report questionnaires and attend a face-to-face visit at the Hospital Universitari Sant Joan de Reus (Reus, Tarragona, Spain) to collect biological samples. Blood and semen samples were then collected on the same day.

### Ethical approval

The study protocol was approved by the Institut d’Investigació Sanitària Pere Virgili Ethics Committee (Reference: CEIM: 181/2019) and the study was conducted according to the ethical standards laid down in the Declaration of Helsinki. All participants involved in the study signed to give written informed consent.

### Sperm quality parameters

Conventional complete semen quality analyses were performed after 3–7 days of sexual abstinence following the World Health Organization protocol (World Health Organization, 2010). An Olympus CX43 phase-contrast microscope (Olympus Corporation, Tokyo, Japan) was used in conjunction with the Computer Aided Sperm Analysis (CASA) SCA^®^ System version 6.5.0.67 (Microptic, Barcelona, Spain) to assess microscopic characteristics of spermatozoa. For each seminogram, the following parameters were analyzed: sperm concentration (×10^6^/ml), sperm total count (×10^6^/ejaculated), total motility (%), progressive and non-progressive motility (%), vitality (%), and normal morphology (%). Motility, vitality, and morphology parameters were evaluated in 200 spermatozoa. Sperm vitality was assessed through the hypoosmotic swelling test (HOS test) and sperm morphology was evaluated using the Hemacolor (Merck KGaA, Darmstadt, Germany) staining protocol. Subsequently, aliquots of 15 M/ml of sperm cells were frozen at −80°C until used.

### Sperm and leukocyte genomic DNA isolation

Frozen peripheral blood leukocytes and sperm samples were used to extract genomic DNA using the DNeasy Blood & Tissue Kit (QIAGEN, Hilden, Germany) according to the manufacturer’s recommendations. Quantity and quality of the isolated sperm and leukocyte DNA were analyzed using a NanoDrop 2000 spectrophotometer (Thermo Fisher Scientific, Waltham, MA, USA).

### Methylation analysis

To evaluate somatic cell contamination on sperm samples, a DNA methylation analysis was performed using the *DLK1* locus as quality control in a subsample of 60 participants. This region contains CpGs which are differentially methylated between somatic and sperm cells; these regions have low methylation levels in spermatozoa compared to somatic cells. For this analysis, isolated sperm DNA was bisulphite-converted following the instructions from the EZ DNA Methylation Kit (Zymo Research, Irvine, CA, USA) and sequenced using the Infinium MethylationEPIC v2.0 BeadChip Kit array (Illumina, San Diego, CA, USA). A positive control was included using peripheral blood mononuclear cells (PBMCs) from 102 randomly selected participants from the AIRWAVE study that were analyzed using the Infinium MethylationEPIC v1.0 BeadChip Kit array. The β-values from the methylation arrays were processed using the software package minfi for R. β-values were obtained according to the methylation intensity at each CpG region as: β-values=methylated/(methylated+unmethylated), ranging from 0 to 1 (0 unmethylated; 1 completely methylated).

### Telomere length determination

TL in sperm and leukocytes was measured by quantitative real-time polymerase chain reaction (qPCR) following a protocol adapted from O’Callaghan and Fenech (2011). The relative quantification of telomeres (T) was conducted using a single-copy gene (S, human 36B4) as a reference for each sample and results were expressed as telomere to single-copy gene ratio (T/S ratio). To complete a final volume of 20 µl per well, 9 µl of leukocyte or sperm DNA (5 ng/µl), 10 µl of SYBR Select Master Mix (Thermo Fisher Scientific, Waltham, MA, USA), and 1 µl of primers (Merck KGaA, Darmstadt, Germany) were used. Samples were amplified in two PCRs: the first was to determine the cycle threshold (Ct) value for telomere (T) amplification, and the second was to determine the Ct value for the 36B4 single-copy gene using the corresponding primers. In addition, a six-point standard curve was included in each plate using 10-fold dilutions of known quantities of standards of telomere and 36B4 (linearity agreement *R*^2^ > 0.97). The quantification of the relative copy numbers (T/S) was performed in triplicates using 96-well plates in a CFX96 Touch Real-Time PCR Detection System (Bio-Rad Laboratories, Hercules, CA, USA). Primer and standard sequences for telomeres and 36B4 are detailed elsewhere (O’Callaghan and Fenech, 2011). Leukocyte and sperm DNA samples from the same participant were run in the same plate. Intra- and inter-assay coefficients of variability were calculated and when the results were above 10%, the samples were reanalyzed.

### Covariates

General socio-demographic (age, educational level) and lifestyle information (diet, physical activity, and smoking status) were collected by online questionnaires. Anthropometric measurements (weight, height, and waist circumference) were recorded with the participants wearing light clothing and without shoes. BMI (kg/m^2^) was calculated by dividing weight (kg) by the square of the height (m). For physical activity information, participants completed the validated short version of the REGICOR (Registre Gironi del Cor) Physical Activity Questionnaire (Metabolic Equivalent of Task (MET)—min/week) (Molina *et al.*, 2017). Dietitians used a validated food frequency questionnaire to estimate dietary food consumption (Fernández-Ballart *et al.*, 2010). The responses for each food item were then converted into daily grams, using the standard portion size of each food. Total daily energy was then calculated using the e-Diet Base URV^®^.

### Statistical analyses

We used the January 2024 Led-Fertyl database. The Kolmogorov–Smirnov test was used to assess the normal distribution of the variables. Sperm and leukocyte TL and total sperm count, concentration, and normal morphology were cubic root-transformed to approach normality. For general characteristics and sperm-related parameters, mean and SD or median [P25–P75] for continuous variables and number (percentage, %) for categorical variables were reported. Differences across tertiles of sperm or leukocyte TL were analyzed by one-way ANOVA or Kruskal–Wallis test according to normal or skewed distributions, respectively. To compare categorical variables across TL tertiles, the chi-squared test was used.

In the present analysis, sperm and leukocyte TL, in continuous and in tertiles, were defined as the exposures, and sperm quality parameters were defined as outcomes. Multivariable linear regression models adjusted for several potential confounders were fitted to test the associations between sperm and leukocyte TL and sperm concentration, total count, total motility, progressive and non-progressive motility, vitality, and normal morphology. Tertile 1, which corresponds to the shortest TL, was used as a reference. Model 1 was adjusted for age (years), BMI (kg/m^2^), and sexual abstinence (days). Model 2 (fully adjusted model) was further adjusted for energy intake (kcal/day), physical activity (METs—min/week), smoking status (never, former, current), and educational level (high school or less, college or high education).

As a sensitivity analysis, participants with sperm concentration and/or total count below the reference lower limits according to the WHO 2010 guidelines (World Health Organization, 2010) were excluded from the analysis of the associations between sperm/leukocyte TL and sperm parameters.

A *P*-value <0.05 was considered statistically significant and the Stata 14.2 software program (StataCorp LP, College Station, TX, USA) was used for all analyses.

## Results

**Figure 1.**
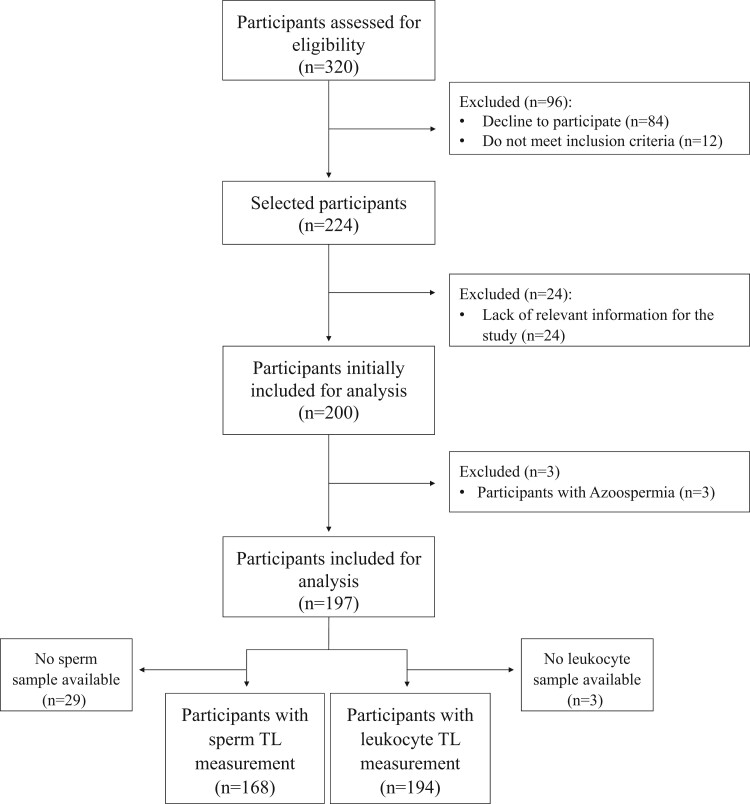
**Flowchart of Led-Fertyl study participants included in the present analysis**. TL, telomere length.

Figure 1 depicts the complete flowchart of the study population included in the present analysis. From a total of 200 participants initially included, three participants were excluded because of azoospermia. Sperm and leukocyte TL were measured in 168 and 194 participants, respectively.

General characteristics and sperm quality parameters of the 197 included Led-Fertyl participants are shown in Table 1. The median age of the participants was 28 years, and the mean BMI was 24.4 kg/m^2^. Overall, 69% of the volunteers were non-smokers and a 64% had a high educational level. In Tables 2 and 3, the same general characteristics according to tertiles of sperm and leukocyte TL are reported, respectively. Compared to participants in the lowest sperm and leukocyte TL tertile, those in the top tertile had higher sperm concentrations and total counts.

**Table 1.** General characteristics of the study population.

Characteristics	n	
Age (years)	197	28 [24–32]
Anthropometric measurements		
Weight (kg)	197	75.5 [70–81.7]
Waist circumference (cm)	197	81.9 [77.2–87.6]
BMI (kg/m²)	197	24.4 (3.2)
Energy intake (kcal/day)	197	2649.7 (635.4)
Leisure-time physical activity (METs—min/week)	197	3572.9 [1767.8–5307.7]
Educational level, n (%)	197	
High school or less		71 (36)
College or high education		129 (64)
Smoking status, n (%)	197	
Current		24 (12.2)
Former		25 (12.7)
Never		136 (69)
Not reported		12 (6.1)
Seminogram parameters		
Sexual abstinence (days)	197	4 [3–5]
Semen volume (ml)	197	3.5 [2.5–4.5]
Sperm concentration (×10^6^/ml)	197	48.6 [29–84.6]
Total sperm count (×10^6^)	197	168.4 [98.8–283.8]
Motility	197	
Total motility (%)		60.1 (16.4)
No motility (%)		39.6 (16.2)
Vitality (%)	196	81.5 [75.8–88.5]
Normal sperm morphology (%)	196	9.3 [5–15]
Sperm TL (T/S ratio)	168	3.2 [2.3–4.8]
Leukocyte TL (T/S ratio)	194	1.5 [1.1–2]

Data are expressed as mean and SD or median and [25th–75th] percentile for continuous variables and number and percentage for categorical variables.

BMI, body mass index; MET, metabolic equivalent of task; TL, telomere length; T/S, telomere to single-copy gene.

**Table 2.** General characteristics of the study population according to tertiles of sperm telomere length.

	Sperm telomere length (T/S ratio)			
	T1	T2	T3	
	(n = 56)	(n = 56)	(n = 56)	*P*-value
Age (years)	27 [24–32]	29 [24.5–32]	28.5 [23–33]	0.553
Anthropometric measurements				
Weight (kg)	76.2 [71.5–80.1]	74.9 [68.8–80.3]	76.7 [68.8–81.6]	0.663
Waist circumference (cm)	81.9 [77.1–86]	81.6 [77.5–85.3]	81.5 [76.7–87.6]	0.933
BMI (kg/m²)	24.4 (3.3)	23.8 (2.7)	24.5 (2.8)	0.278
Energy intake (kcal/day)	2658.8 (724.5)	2606.2 (569.3)	2661.7 (593)	0.872
Leisure-time physical activity (METs—min/week)	3123.5 [1866.4–4631.7]	3779.5 [1841.5–5324]	3584.1 [1591.6–5489.5]	0.585
Educational level, n (%)				0.832
High school or less	21 (37.5)	19 (33.9)	18 (32.1)	
College or high education	35 (62.5)	37 (66.1)	38 (67.9)	
Smoking status, n (%)				0.040
Current	5 (8.9)	4 (7.1)	11 (19.6)	
Former	8 (14.3)	10 (17.9)	2 (3.6)	
Never	38 (67.9)	41 (73.2)	37 (66.1)	
Not reported	5 (8.9)	1 (1.8)	6 (10.7)	
Seminogram parameters				
Sexual abstinence (days)	4 [3–5]	4 [3–4.8]	4 [3–5]	0.559
Semen volume (ml)	3.8 [2.9–5]	3.6 [2.7–4.8]	3.5 [2.9–4.5]	0.874
Sperm concentration (×10^6^/ml)	40.1 [28.8–66.1]	62.1 [35.9–91.8]	66.5 [47.9–109.3]	<0.001
Total sperm count (×10^6^)	135.5 [102.8–226]	194 [127.8–303]	253.7 [171.9–361]	<0.001
Motility				
Total motility (%)	64 (13.1)	63.3 (16.4)	60.6 (15.9)	0.224
No motility (%)	35.9 (13.2)	35.9 (15.4)	39.4 (15.9)	0.352
Vitality (%)	81 [72.5–88.5]	83.8 [77–89.8]	80 [75.8–87]	0.167
Normal sperm morphology (%)	11.5 [6–19]	11 [5–15]	7.8 [4.8–14]	0.131
Leukocyte TL (T/S ratio)	1.2 [0.9–1.6]	1.6 [1.2–1.9]	2 [1.5–2.6]	<0.001

Data are expressed as mean and SD or median and [25th–75th] percentile for continuous variables and number and percentage for categorical variables. Differences across tertiles of sperm TL were analyzed by one-way ANOVA or Kruskal–Wallis test according to normal or skewed distributions, respectively. To compare categorical variables across TL tertiles, the chi-squared test was used.

BMI, body mass index; MET, metabolic equivalent of task; T, tertile; TL, telomere length; T/S, telomere to single-copy gene.

**Table 3.** General characteristics of the study population according to tertiles of leukocyte telomere length.

	Leukocyte telomere length (T/S ratio)			
	T1	T2	T3	
	(n = 65)	(n = 65)	(n = 64)	*P*-value
Age (years)	29 [24–33]	29 [24–31]	28 [23–33]	0.878
Anthropometric measurements				
Weight (kg)	76 [73–83.5]	75.2 [68.5–81.7]	75.8 [68.3–81.1]	0.251
Waist circumference (cm)	83 [78.3–89.4]	81 [77–84.5]	81.7 [76.8–87.3]	0.139
BMI (kg/m²)	25.2 (3.5)	23.9 (2.9)	24 (2.9)	0.298
Energy intake (kcal/day)	2566.8 (646.1)	2656.2 (621.3)	2699.3 (623.6)	0.477
Leisure-time physical activity (METs—min/week)	3729.1 [1965–5137.5]	3615.4 [1864.8–5757.6]	3128.7 [1521.7–5311.2]	0.509
Educational level, n (%)				0.244
High school or less	20 (30.8)	21 (32.3)	28 (43.8)	
College or high education	45 (69.2)	44 (67.3)	36 (56.2)	
Smoking status, n (%)				0.571
Current	7 (10.8)	7 (10.8)	9 (14.1)	
Former	11 (16.9)	9 (13.9)	5 (7.8)	
Never	43 (66.1)	47 (72.3)	44 (68.8)	
Not reported	4 (6.2)	2 (3.1)	6 (9.4)	
Seminogram parameters				
Sexual abstinence (days)	3 [3–5]	4 [3–5]	4 [3–5]	0.189
Semen volume (ml)	3.2 [2.3–4]	3.5 [2.7–5]	3.7 [2.8–4.5]	0.100
Sperm concentration (×10^6^/ml)	42 [21.5–74.2]	43.5 [25.4–65.7]	63.7 [37.6–111.8]	0.001
Total sperm count (×10^6^)	130.1 [64.3–230.1]	141.9 [83.8–232.7]	243.9 [137.9–350.7]	<0.001
Motility				
Total motility (%)	59.1 (17.5)	58.8 (16.5)	62.5 (15.3)	0.562
No motility (%)	40.8 (17.6)	40.9 (16.1)	37 (15.1)	0.486
Vitality (%)	80.8 [73–87.8]	85.5 [76–91]	80.8 [75.8–85.8]	0.112
Normal sperm morphology (%)	8 [4.5–15]	10 [5–16.5]	9.5 [5.5–14.5]	0.841
Sperm TL (T/S ratio)	2.2 [1.4–3.1]	3.2 [2.3–4.5]	4.2 [3.2–5.6]	<0.001

Data are expressed as mean and SD or median and [25th–75th] percentile for continuous variables and number and percentage for categorical variables. Differences across tertiles of leukocyte TL were analyzed by one-way ANOVA or Kruskal–Wallis test according to normal or skewed distributions, respectively. To compare categorical variables across TL tertiles, the chi-squared test was used.

BMI, body mass index; MET, metabolic equivalent of task; T, tertile; TL, telomere length; T/S, telomere to single-copy gene.

The *DLK1* locus quality control confirmed that the sperm subsamples analyzed were clear of somatic cell contamination (Supplementary Fig. S1).

**Figure 2.**
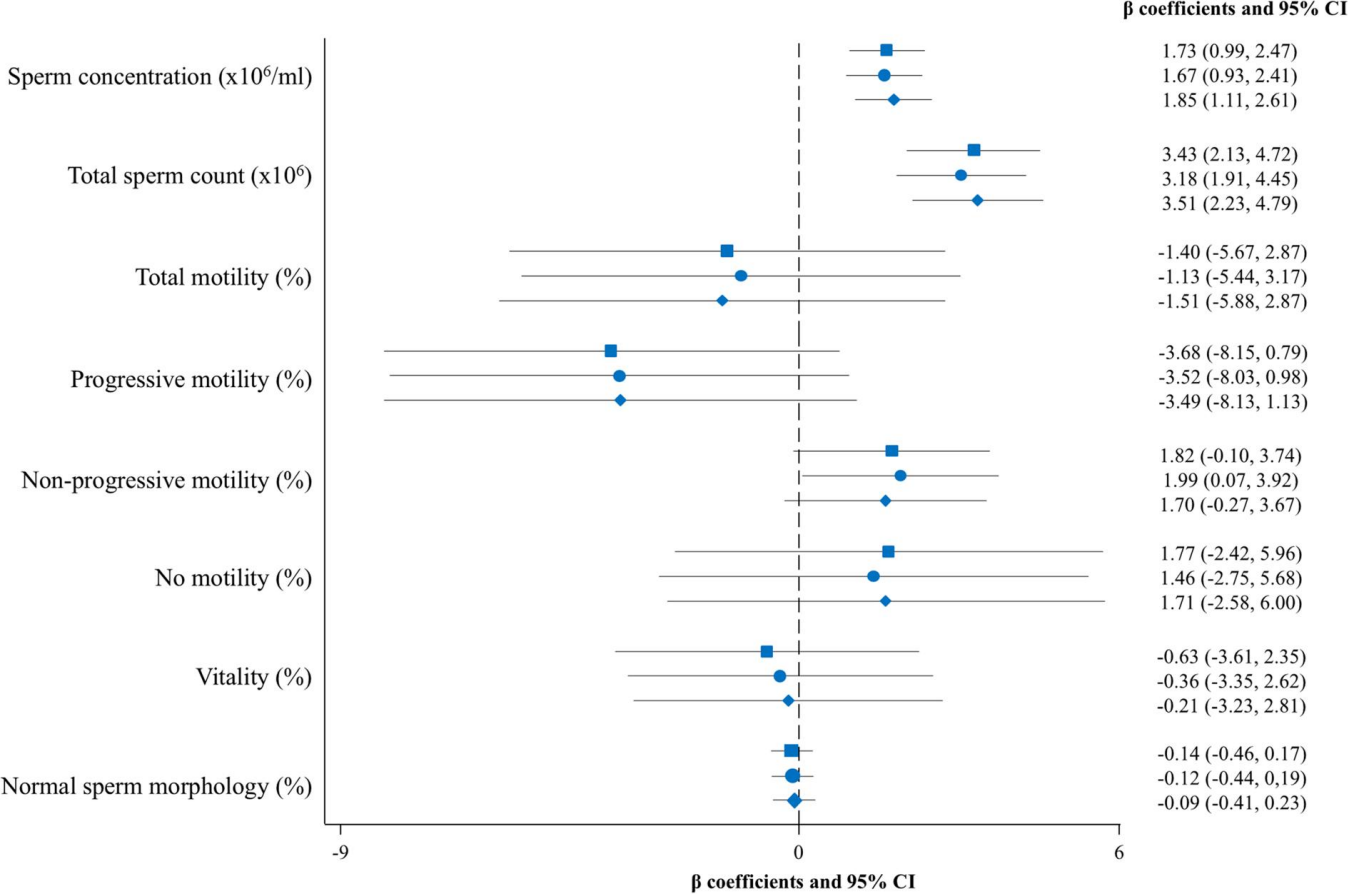
**Multivariable-adjusted β coefficients and 95% CI of the associations between sperm telomere length as a continuous variable and sperm parameters**. The crude model is represented with a square. Model 1 is represented with a circle and was adjusted for age (years), body mass index (kg/m^2^), and abstinence (days). Model 2 is represented with a rhombus and was further adjusted for energy intake (kcal/day), physical activity (METs—min/week), smoking status (never, former, current), and educational level (high school or less, college or high education). Sperm telomere length, total sperm count, and concentration, and normal morphology were cubic root-transformed to approach normality. MET, metabolic equivalent of task.

Table 4 and Fig. 2 summarize the multivariable-adjusted β coefficients (95% CI) of the linear regression analysis evaluating the cross-sectional associations between sperm TL and sperm quality parameters. Compared to participants in the reference tertile (T1) of sperm TL, those in tertiles 2 (T2) and 3 (T3) showed a higher sperm concentration (fully adjusted model, β: 1.09; 95% CI: 0.09–2.09 and β: 2.06; 95% CI: 1.04–3.09 for T2 and T3, respectively; *P*-trend <0.001). Additionally, participants in the highest tertile of sperm TL had a higher total sperm count (fully adjusted model, β: 3.83; 95% CI: 2.08–5.58 for T3 vs T1; *P*-trend <0.001) (Table 4). These positive associations with sperm concentration and total count were also found when sperm TL was modelled as a continuous variable (Fig. 2). When sensitivity analyses were performed by excluding 5 out of 168 participants with sperm concentration and/or total count below the reference lower limits, the observed associations remained significant.

**Table 4.** Multivariable-adjusted β coefficients and 95% CIs of sperm parameters across tertiles of sperm telomere length.

	Sperm telomere length (T/S ratio)			
	T1	T2	T3	
	(n = 56)	(n = 56)	(n = 56)	*P*-trend
**Sperm telomere length** ^a^	[0.26–1.59]	[1.59–2.02]	[2.02–3.06]	
**Sperm concentration (×10^6^/ml)** ^a^				
Crude model	0 (Ref.)	**1.11 (0.11, 2.12)**	**1.91 (0.91, 2.92)**	**<0.001**
Model 1	0 (Ref.)	**1.02 (0.01, 2.03)**	**1.85 (0.83, 2.86)**	**<0.001**
Model 2	0 (Ref.)	**1.09 (0.09, 2.09)**	**2.06 (1.04, 3.09)**	**<0.001**
**Total sperm count (×10^6^)** ^a^				
Crude model	0 (Ref.)	1.35 (−0.41, 3.12)	**3.75 (1.98, 5.52)**	**<0.001**
Model 1	0 (Ref.)	1.11 (−0.61, 2.85)	**3.39 (1.65, 5.13)**	**<0.001**
Model 2	0 (Ref.)	1.12 (−0.58, 2.84)	**3.83 (2.08, 5.58)**	**<0.001**
**Total motility (%)**				
Crude model	0 (Ref.)	−0.67 (−6.33, 4.99)	−3.36 (−9.02, 2.30)	0.234
Model 1	0 (Ref.)	−0.63 (−6.34, 5.07)	−2.97 (−8.69, 2.75)	0.298
Model 2	0 (Ref.)	−0.07 (−5.77, 5.63)	−3.79 (−9.61, 2.00)	0.193
**Progressive motility (%)**				
Crude model	0 (Ref.)	−2.88 (−8.80, 3.02)	−6.25 (−12.16, −0.33)	**0.038**
Model 1	0 (Ref.)	−3.02 (−9.00, 2.94)	−5.92 (−11.91, 0.05)	0.052
Model 2	0 (Ref.)	−2.67 (−8.71, 3.36)	−6.06 (−12.21, 0.08)	0.052
**Non-progressive motility (%)**				
Crude model	0 (Ref.)	**3.18 (0.65, 5.72)**	**2.64 (0.11, 5.18)**	0.051
Model 1	0 (Ref.)	**3.35 (0.82, 5.89)**	**2.84 (0.30, 5.38)**	**0.037**
Model 2	0 (Ref.)	**3.48 (0.93, 6.03)**	2.31 (−0.28, 4.90)	0.091
**No motility (%)**				
Crude model	0 (Ref.)	−0.01 (−5.56, 5.55)	3.45 (−2.11, 9.01)	0.210
Model 1	0 (Ref.)	−0.04 (−5.63, 5.55)	2.98 (−2.61, 8.58)	0.281
Model 2	0 (Ref.)	−0.53 (−6.13, 5.05)	3.61 (−2.08, 9.30)	0.206
**Vitality (%)**				
Crude model	0 (Ref.)	2.36 (−1.55, 6.28)	−0.77 (−4.69, 3.14)	0.638
Model 1	0 (Ref.)	2.53 (−1.39, 6.46)	−0.39 (−4.33, 3.53)	0.778
Model 2	0 (Ref.)	2.24 (−1.66, 6.16)	−0.24 (−4.23, 3.74)	0.878
**Normal sperm morphology (%)** ^a^				
Crude model	0 (Ref.)	−0.18 (−0.60, 0.23)	**−0.46 (−0.87, −0.04)**	**0.030**
Model 1	0 (Ref.)	−0.18 (−0.6, 0.24)	**−0.43 (−0.64, −0.01)**	**0.045**
Model 2	0 (Ref.)	−0.24 (−0.66, 0.18)	−0.37 (−0.79, 0.05)	0.085

Multivariable linear regression models were fitted to test the associations between sperm telomere length (TL) and sperm parameters. Tertile 1 (T1), which corresponds to the shortest telomere length, was used as a reference. Model 1: adjusted for age (years) and body mass index (BMI; kg/m^2^) and abstinence (days). Model 2: further adjusted for energy intake (kcal/day), physical activity (METs—min/week), smoking status (never, former, current), educational level (high school or less, college or high education).

a Sperm TL, total sperm count, concentration, and normal morphology were cubic root-transformed to approach normality. Bold indicates *P*-value or *P*-trend *<* 0.05.

**Figure 3.**
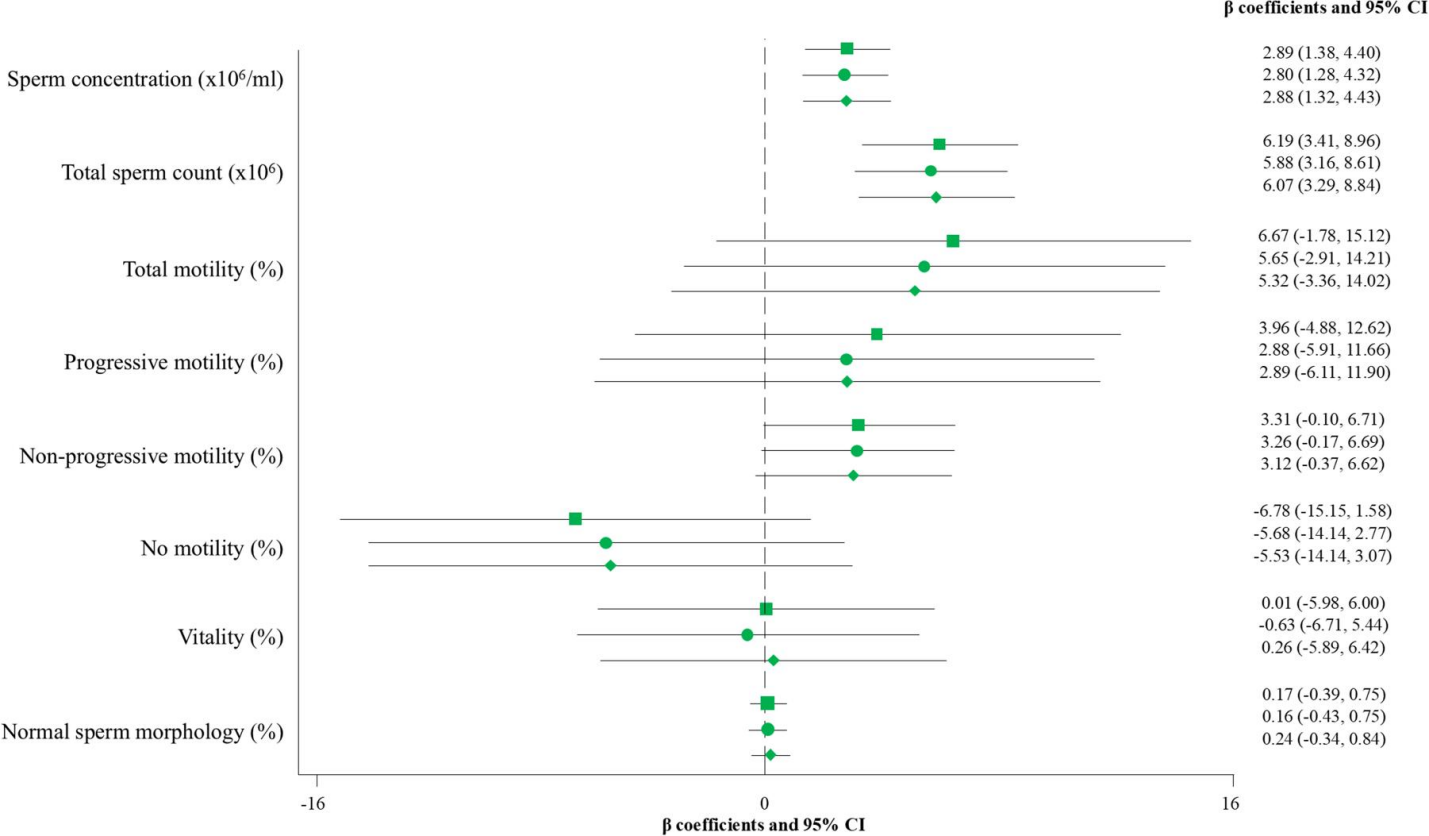
**Multivariable-adjusted β coefficients and 95% CI of the associations between leukocyte telomere length as a continuous variable and sperm parameters**. The crude model is represented with a square. Model 1 is represented with a circle and was adjusted for age (years), body mass index (kg/m^2^), and abstinence (days). Model 2 is represented with a rhombus and was further adjusted for energy intake (kcal/day), physical activity (METs—min/week), smoking status (never, former, current), and educational level (high school or less, college or high education). Leukocyte telomere length, total sperm count and concentration, and normal morphology were cubic root-transformed to approach normality. MET, metabolic equivalent of task.

Similar results were observed in relation to leukocyte TL. Participants in the top tertile of leukocyte TL showed higher sperm concentration (fully adjusted model, β: 1.49; 95% CI: 0.44–2.54 for T3 vs T1; *P*-trend = 0.004) and total count (fully adjusted model, β: 3.49; 95% CI: 1.62–5.35 for T3 vs T1; *P*-trend <0.001) compared to participants in T1 (Table 5). Moreover, when leukocyte TL was expressed as a continuous variable in the analysis, positive associations between leukocyte TL and sperm concentration and sperm count were also identified (Fig. 3). A total of 21 out of 194 participants had sperm concentrations and/or total counts below the reference lower limits. Upon exclusion of these participants, the results were consistent.

**Table 5.** Multivariable-adjusted β coefficients and 95% CIs of sperm parameters across tertiles of leukocyte telomere length.

	Leukocyte telomere length (T/S ratio)			
	T1	T2	T3	
	(n = 65)	(n = 65)	(n = 64)	*P*-trend
**Leukocyte telomere length** ^a^	[0.64–1.10]	[1.10–1.33]	[1.33–2.24]	
**Sperm concentration (×10^6^/ml)** ^a^				
Crude model	0 (Ref.)	−0.36 (−1.39, 0.65)	**1.56 (0.51, 2.57)**	**0.002**
Model 1	0 (Ref.)	−0.41 (−1.43, 0.61)	**1.45 (0.42, 2.48)**	**0.004**
Model 2	0 (Ref.)	−0.35 (−1.33, 0.67)	**1.49 (0.44, 2.54)**	**0.004**
**Total sperm count (×10^6^)** ^a^				
Crude model	0 (Ref.)	−0.40 (−2.25, 1.44)	**3.71 (1.85, 5.58)**	**<0.001**
Model 1	0 (Ref.)	−0.49 (−2.31, 1.33)	**3.39 (1.51, 5.22)**	**<0.001**
Model 2	0 (Ref.)	−0.43 (−2.18, 1.40)	**3.49 (1.62, 5.35)**	**<0.001**
**Total motility (%)**				
Crude model	0 (Ref.)	−0.34 (−6.04, 5.34)	3.39 (−2.32, 9.10)	0.224
Model 1	0 (Ref.)	−1.15 (−6.92, 4.61)	2.59 (−3.22, 8.38)	0.342
Model 2	0 (Ref.)	−0.95 (−6.67, 4.82)	2.32 (−3.64, 8.11)	0.423
**Progressive motility (%)**				
Crude model	0 (Ref.)	−1.69 (−7.51, 4.11)	2.27 (−3.56, 4.11)	0.405
Model 1	0 (Ref.)	−2.59 (−8.52, 3.30)	1.40 (−4.57, 7.34)	0.571
Model 2	0 (Ref.)	−2.37 (−8.16, 3.58)	1.33 (−4.72, 7.39)	0.609
**Non-progressive motility (%)**				
Crude model	0 (Ref.)	1.57 (−0.70, 3.90)	1.99 (−0.31, 4.29)	0.098
Model 1	0 (Ref.)	1.59 (−0.75, 3.87)	1.98 (−0.34, 4.31)	0.105
Model 2	0 (Ref.)	1.53 (−0.78, 3.86)	1.83 (−0.52, 4.20)	0.138
**No motility (%)**				
Crude model	0 (Ref.)	0.08 (−5.54, 5.72)	−3.49 (−9.45, 1.85)	0.171
Model 1	0 (Ref.)	0.97 (−4.77, 6.66)	−2.94 (−8.68, 2.78)	0.277
Model 2	0 (Ref.)	0.75 (−4.95, 6.46)	−2.69 (−8.51, 3.11)	0.333
**Vitality (%)**				
Crude model	0 (Ref.)	3.17 (−0.83, 7.18)	0.27 (−3.41, 4.29)	0.988
Model 1	0 (Ref.)	2.79 (−1.28, 6.86)	−0.13 (−4.22, 3.96)	0.843
Model 2	0 (Ref.)	2.82 (−1.26, 6.89)	0.44 (−3.69, 4.58)	0.919
**Normal sperm morphology (%)** ^a^				
Crude model	0 (Ref.)	0.09 (−0.21, 0.48)	0.10 (−0.28, 0.49)	0.597
Model 1	0 (Ref.)	0.07 (−0.32, 0.47)	0.09 (−0.30, 0.49)	0.644
Model 2	0 (Ref.)	0.07 (−0.32, 0.47)	0.15 (−0.25, 0.55)	0.465

Multivariable linear regression models were fitted to test the associations between leukocyte telomere length (TL) and sperm parameters. Tertile 1 (T1), which corresponds to the shortest telomere length, was used as a reference. Model 1: adjusted for age (years) and body mass index (BMI; kg/m^2^) and abstinence (days). Model 2: further adjusted for energy intake (kcal/day), physical activity (METs—min/week), smoking status (never, former, current), educational level (high school or less, college or high education).

a Leukocyte TL, total sperm count, concentration, and normal morphology were cubic root-transformed to approach normality. Bold indicates *P*-value or *P*-trend *<* 0.05.

No associations were identified in the fully adjusted models between sperm or leukocyte TL and the other sperm quality parameters considered (total, progressive, and non-progressive motility, vitality, and normal morphology), neither across TL tertiles (*P* for trends >0.05) nor as continuous variables.

## Discussion

In the present cross-sectional study, longer sperm and leukocyte TL were positively associated with higher sperm concentration and total count in a general population of healthy men of reproductive age, independent of age, BMI, and other potential confounding factors. The results were consistent regardless of whether TL was modelled as tertiles or as a continuous variable.

The study of the association between TL and sperm quality and male infertility is crucial since short telomeres have been associated with impaired sperm parameters. As reported in our recently published meta-analysis, shorter sperm TL was found in men with oligozoospermia or infertility, compared to fertile or normozoospermic individuals (Fernández de la Puente *et al.*, 2024). In individuals with other altered sperm quality conditions such as teratozoospermia (i.e. over 96% of spermatozoa with abnormal morphology), TL in spermatozoa was also shown to be shorter compared to healthy donors (Fattahi *et al.*, 2023). Consistent with our meta-analysis, other recent studies have shown that sperm and leukocyte TL were shorter in oligospermic men compared to fertile men (Dhillon *et al.*, 2024; Margiana *et al.*, 2024). These results clearly indicate that sperm parameters are compromised when the mechanisms for maintaining or elongating TL are impaired. This situation has been shown to be accompanied by processes such as increased concentrations of oxidative species and the expression of pro-apoptotic markers that could partially lead to reduced sperm quality and, consequently, male infertility (Margiana *et al.*, 2024).

In the present analysis including healthy volunteer men of reproductive age, even after excluding participants who had a sperm concentration and total count below the reference limit, the positive associations found between sperm and leukocyte TL and sperm count and concentration remained consistent. Our results are in line with the positive correlations found between TL and the same seminogram parameters identified in recent research (Dhillon *et al.*, 2024). Moreover, in that study, no correlations were reported between TL measured in both cell types and sperm total motility or morphology (Dhillon *et al.*, 2024), which is also in line with our findings. These results suggest that the length of telomeres from spermatozoa and leukocyte cells could be used as a potential biomarker of sperm quality in terms of sperm concentration and total count. Further studies are warranted in the future to elucidate the relationship between TL and the remaining sperm parameters.

The evidence pertaining to the relationship between longer telomeres in spermatozoa and reproductive outcomes has yielded contradictory results. The positive association between sperm TL and the percentage of fertilization rates found in one study (Berneau *et al.*, 2020) was not replicated by other authors (Yang *et al.*, 2015; Yuan *et al.*, 2022), and the same occurred with embryological parameters (Yang *et al.*, 2015; Berneau *et al.*, 2020). In a meta-analysis, longer sperm TL was associated with higher rates of clinical pregnancy (Yuan *et al.*, 2022). In addition, paternal age has been associated with longer leukocyte TL from the offspring (Kimura *et al.*, 2008), suggesting a potential hereditary component. Longer telomeres at birth could contribute to healthier ageing trajectories throughout life. However, the exact mechanisms and implications of sperm TL on fertilization and offspring inheritance are still an active area of research.

Telomeres, in conjunction with a complex of proteins, attach to the nuclear envelope forming a cluster during the prophase I step in meiosis. This process could facilitate homologous chromosome alignment for recombination, which is a crucial stage in completing meiosis and ensuring the proper development of germ cells (reviewed in Handel and Schimenti, 2010). Whether telomeres themselves are involved in these meiotic processes is still unknown. Spermatocytes from idiopathic infertile men exhibit reduced meiotic crossover rates and an alteration in the association between the telomeres and the RNAs that maintain the integrity of the telomeric structure (Reig-Viader *et al.*, 2014). Thus, failed recombination, in combination with an alteration of telomeric homeostasis, could be a significant factor in reducing sperm quality and, consequently, increasing the risk of infertility.

Based on previous evidence and the findings reported here, it seems that the physiological function of telomeres and the proteins responsible for maintaining their length and integrity are crucial for preventing segregation errors and ensuring proper spermatogenesis.

### Strengths and limitations of the study

Considerable limitations should be taken into account in the interpretation of our findings. Firstly, the cross-sectional design of the study makes it impossible to establish a cause-effect relationship. Although it was a challenge to recruit 200 volunteers, the sample size cannot be considered large. The main strength of the Led-Fertyl study is the inclusion of a general population of reproductive age, consisting of young volunteers in good health state who did not attend fertility clinics. Secondly, the wide range of information collected on sociodemographic and lifestyle factors enabled us to control for several confounders in all analyses. However, it is important to note that residual confounding factors cannot be completely ruled out. Additionally, semen samples were processed using a standardized protocol through the CASA SCA^®^ system, thereby reducing any potential subjectivity.

## Conclusion

In light of our findings, it can be concluded that sperm and leukocyte TL are associated with sperm parameters in terms of concentration and total count. Reduced TL may partially explain cases of impaired spermatogenesis. Nevertheless, additional determinations based on oxidative stress, protein activity, and expression of telomere dynamic-related genes can supplement these findings and clarify the mechanisms underlying these associations. Further studies with a larger sample size are necessary to confirm our results and investigate the further implications.

## Supplementary Material

hoae062_Supplementary_Data

## Data Availability

The data are not publicly available outside of the core Led-Fertyl research group, owing to data regulations and ethical considerations. This precaution has been taken to safeguard the consent of research participants, as their original agreement was limited to the utilization of their data by the initial research team. However, the researchers will follow a controlled data-sharing collaboration for research related to the project’s aims. Therefore, investigators who are interested in the present study can contact the senior authors (nancy.babio@urv.cat, jordi.salas@urv.cat) by sending a formal letter of request.
