# Supplementary material for: Sperm and leukocyte telomere length are related to sperm quality parameters in healthy men from the Led-Fertyl study
Source: Hum Reprod Open. 2024 Oct 14;2024(4):hoae062. doi: 10.1093/hropen/hoae062 (PMC11520404; doi:10.1093/hropen/hoae062)
Supplement: hoae062_Supplementary_Data [file hoae062_supplementary_data.pdf]

DLK1 PROMOTER CpGs

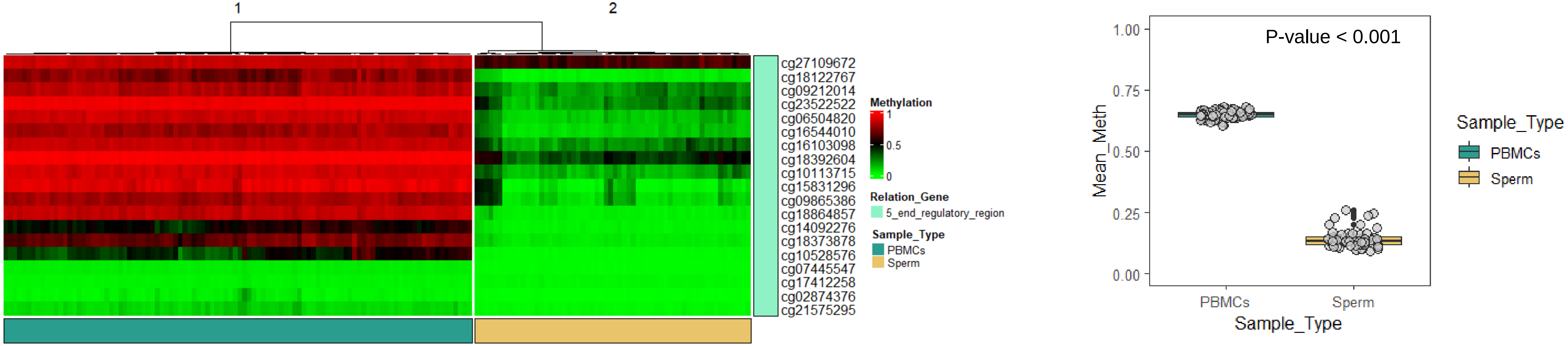

Supplementary Figure S1. Methylation analysis of the *DLK1* locus for quality control of somatic cell contamination. CpG promoter sites associated to *DLK1* gene (HeatMap on the left) and mean methylation values (BoxPlot on the right) in peripheral blood mononuclear cells (PBMCs) and sperm cells are shown. These sites are differentially methylated between PBMCs and sperm cells. The methylation pattern showed no somatic cell contamination in the subsample analyzed.
